# Using Qualitative Methods to Understand the Interconnections Between Cities and Health: A Methodological Review

**DOI:** 10.3389/phrs.2024.1606454

**Published:** 2024-04-08

**Authors:** José Pedro Silva, Ana Isabel Ribeiro

**Affiliations:** ^1^ EPIUnit—Instituto de Saúde Pública da Universidade do Porto, Porto, Portugal; ^2^ Laboratório para a Investigação Integrativa e Translacional em Saúde Populacional (ITR), Porto, Portugal; ^3^ Instituto de Sociologia da Universidade do Porto, Porto, Portugal; ^4^ Departamento de Ciências da Saúde Pública e Forenses e Educação Médica, Faculdade de Medicina da Universidade do Porto, Porto, Portugal

**Keywords:** public health, urban health, qualitative methods, interviews, observation, photovoice, focus groups, mental mapping

## Abstract

**Objective:** Using different perspectives and methods to investigate the links between the urban phenomenon and health is critical in an urbanizing world. This review discusses qualitative methods in the context of urban health research.

**Methods:** We conducted a narrative review following these steps: We identified the qualitative data collection, analysis and sampling methods that could be more relevant for the problems researched in the urban health field. We conducted searches for methodological articles and other documents about those methods. We included some influential materials and examples of empirical urban health studies using those methods.

**Results:** We included 88 studies and identified several qualitative data gathering, analysis and sampling methods relevant for urban health researchers. We present those methods, focusing their strengths and limitations, and providing examples of their use in the field of urban health. These methods are flexible and allow in-depth analysis of small samples by collecting and analyzing rich and nuanced data.

**Conclusion:** This article should contribute to a better understanding of how, and when, qualitative methods may improve our knowledge on urban health.

## Introduction

Interest in how urban life shapes health has a long history [1]. 55% of the world’s population lived in cities in 2018, a proportion expected to increase to 68% by 2050. The growth of the urban population in the global South strongly contributes to this increase [2]. Place matters for health [3], therefore, the study of the complicated connections between the urban environment and health is a relevant issue.

There have been calls for urban health research combining the approaches of different disciplines [1] and methodologies, including qualitative methods [3–6]. These may contribute to the understanding of urban health, defined as the study of how urban life contributes to shape human health, often complementing quantitative results. Both types of methods are valid on their own, but may, and have been, combined.

However, there has been an under-utilization of qualitative methods in urban health [7, 8]. Qualitative papers have been excluded from several systematic reviews about the effects of the built environment on human health [9–11], and are still considered lower priority by several leading medical and health journals [12, 13]. Misconceptions about qualitative methods still remain among health and medical researchers [8], who are usually more familiarized with quantitative approaches [13]. As far as we are aware, there are no reviews exploring how qualitative methods may be valuable for this field of inquiry.

We discuss how qualitative techniques of sampling, data collection and data analysis may be used in urban health research. Our focus will not be qualitative research as a whole, nor any specific qualitative tradition of inquiry, such as, for example, grounded theory (an approach seeking to develop theories from data) [14]. Also, we will not focus on epistemological and ontological issues or research design issues. Our stance is a practical one, concerned with methods as tools to achieve research goals (we discuss this stance further ahead). Our aim is not to exhaustively cover qualitative methods. Instead, we will focus on some of the most commonly used methods and on some others, perhaps less common, but that we consider highly valuable for studying urban health. We will provide examples of urban health studies employing these methods, based on our knowledge of the literature. Thus, this review is built on the work of many researchers, but contains choices and judgements that result from our own research experiences, practices and inclinations. We intend to raise awareness for the potentialities of qualitative methods in urban health research and to introduce readers inexperienced in qualitative research to their applications in the field.

Although our focus is on qualitative *methods*, not qualitative *research*, it is useful to provide a definition of the latter. Although there is no single universal definition, a recent review intending to arrive at a precise definition of qualitative research has suggested the following as its central features: 1) makes new distinctions possible, 2) iterative research processes (going back-and-forth from theory to evidence and from analysis to data), 3) close contact with the persons being studied and the materials analyzed, 4) allows deep understanding of phenomena [15]. Moreover, qualitative research can deconstruct prevailing categories, concepts and understandings [16, 17] and generate new theoretical ideas [8].

## Methods

To conduct this review, we established what qualitative methods we should cover, considering their popularity and their usefulness for urban health researchers. We then conducted several searches on google scholar, using the following key-words: “qualitative research,” “qualitative methods,” “observation,” “participant observation,” “interviewing,” “interview,” “qualitative interviewing,” “qualitative interview,” “in-depth interview,” “go-along interview,” “walk-along interview,” “focus groups,” “world café,” “photovoice,” “mental mapping,” “qualitative data analysis,” “qualitative analysis,” “qualitative content analysis,” “thematic analysis,” “document analysis,” “qualitative sampling,” “purposive sampling,” “saturation.” We supplemented this search by inspecting the references list of the selected articles and including some influential materials. Also included were some empirical studies, as examples of the application of those methods in urban health research. We examined a broad set of materials, including books, gray literature and scientific articles. All searches were conducted in English. A total of 88 references were included and summarised in a narrative commentary.

## Results

### Observational Methods

Observational methods may be used qualitatively and quantitatively. We will focus on the former. They allow the collection of data about what people do in the settings where their lives unfold [18] and about those settings [19] by witnessing what happens there. They are arguably underused in health research [20, 21]. Despite their name, one may use the five senses to collect data [19]. Doing observation involves a varying degree of involvement in the activities being observed, from participating as much as possible to being, as far as possible, an external observer. Various intermediate positions are possible [19, 21, 22]. Nevertheless, Keiding, informed by constructionism and systems theory, argues that complete non-participation is impossible, as the observer is always a co-producer of the observed situation [22]. In different words, the presence of the researcher affects the observed events [18, 23].

There might be a concern with staged performances or self-censorship [24]. Some authors argue that researchers must strive to reduce this effect [25], while others accept it not as a limitation, but as a vehicle to knowledge [23] or a potential strength of observation: responses to the presence of researchers are data, while staged behaviors and self-censorship may reveal how people perceive themselves and wish to be perceived [24]. Several authors argue that prolonged presence in a setting will build confidence and familiarity, leading people to behave normally while being knowingly observed given enough time [19, 23, 26]. Prolonged engagement is thus important for data credibility. Furthermore, interacting with people, participating in their activities and asking questions are means to reveal information, offering opportunities to clarify events and to access otherwise inaccessible settings and situations [19, 23]. Observation may also be unstructured or follow a previously designed schedule [20, 21]. Some authors argue for a “child-like” stance, where the observer uses an unstructured approach while seeking to set preconceptions and previous knowledge aside and behave as if knowing nothing about what is being observed [19]. This has been criticized on the basis that researchers cannot observe everything happening in a given setting, nor put themselves aside and pretend they are blank pages: there is always some kind of observation criteria, which should be explicit [22].

As an example, Raap et al [27] used ethnographic methods, including walking interviews and participant observation, together with an open-ended questionnaire to examine how cultural understandings of urban green spaces relate to their health potential. They studied green spaces in three deprived neighborhoods during 3 years, revealing residents’ expectations about their cleanliness and their role as places of play and sociability.

### Interviews

Qualitative interviews are generally designed to allow interviewees to talk at length, in their own terms, about topics of interest. They are a common data collection method, however, criticism has been directed at a purported overreliance of social science research on interviewing [28, 29]. Interviews are considered to generate different kinds of data about the interviewees’ lives and contexts, including behaviors, representations, classification systems, emotions, identities, cultural elements, among others [30]. Interviews are used, among other things, to collect information, reveal tacit knowledge, investigate meaning and stimulate reflexivity [31]. However, this understanding has been challenged in different ways. Critics have pointed out the inconsistencies between self-described and actual behavior [32] and between motivations for behavior and *a posteriori* rationalizations about behavior [33]. They have also discussed how research agendas and categories permeate interviews and coach answers, the convoluted identities that interviewees mobilize when providing answers, and the influence of issues of stake and interest [34]. Some authors advert that interviews may be seen as an unproblematic vehicle to individual views and experiences, which neglects their constitutive and performative dimension, thus potentially celebrating cultural features of a society where interviewing is pervasive [28, 29, 35]. Such criticisms have generated debates about the purposes and value of interviewing and interview data [30, 35–42]. Nevertheless, interviews are still frequently used in ways compatible with the understanding described earlier. For example, interviews have been used to study the connections between gentrification and health. Researchers have interviewed residents in gentrifying areas to investigate how gentrification processes affect the health and wellbeing of people living in gentrifying cities and neighborhoods [43–46].

There are many types of interviews, with different purposes, depth and structuration degree. For example, the widely used semi-structured interview combines structure and flexibility while attempting to reach answer depth. It includes some previously defined open questions, but the interviewer may further explore the views of the interviewee and follow interesting perspectives emerging from the conversation by using meaningful unscripted questions [8].

The go-along interview may be important for urban health: it connects the accounts of interviewees with observational data about their urban environment and how they negotiate it. It consists of an interview that bridges the gap between the observation of spatialized everyday practices and experiences and their subjective accounts [47], conducted while interviewee and interviewer move, either by walking (walk-along interview) or driving (drive-along interview) through a familiar environment (for instance, the neighborhood) [4, 47]. This allows the examination of the interviewee’s accounts on practices and experiences within their spatial context [4], while revealing their taken-for-granted (therefore, tacit) dimensions. It also provides cues about the physical and social characteristics of the neighborhood [47, 48], and may allow the inclusion of a temporal dimension, as interviewees may tell stories about the places where they move during the interview [4]. As an example, Lauwers [49] and co-authors have used walk-along interviews in Brussels to investigate how mental health may relate to neighborhood-level physical and social factors.

### Photovoice

Photovoice [50, 51] is a qualitative participative action research method [52]. It uses photographs to reveal meaning [53], to record the concerns and strengths of communities, to promote collective reflection and discussion about relevant issues, and to take those issues to policymakers in order to promote change [51]. Photographs are simultaneously a means to reveal meaning, to enable collective discussion and reflection and to empower individuals and communities. In photovoice studies, research participants are *participants* in a deeper sense, as they often generate and analyze data, co-construct results, and may be involved in other research moments, such as the dissemination of results. Photovoice has been used to reveal the perspectives of urban dwellers on topics such as the links between gentrification and health [54] and between urban food environments and dietary quality [55, 56]. In these studies, participants generated data by taking photographs and discussing their meaning, and participated in data analysis and dissemination of results. Photovoice may also empower participants by promoting knowledge and awareness of relevant community topics, improving self-perception, and fostering the expansion of social networks [57].

### Mental Mapping

Mental mapping consists of asking research participants to draw and label their own maps or to label existing maps, individually or in group. The method is useful for multiple disciplines [58, 59], but remains under-used [60]. Its focus is on exploring perceptions about space, not cartographic accuracy. Participants may be invited to label preexisting maps or to draw and label a map of a given space as they see it, and to portrait their movement in an average day [58]. These exercises may be individual or collective, and are often accompanied or followed by an interview or focus group, or implemented within an ethnographic inquiry [58]. This method allows to visually tell about space in ways that transcend the expressive possibilities of words alone [58]. It provides insights on the spatial dimension of everyday activities and on the meanings and identities attached to specific places, linking space to its lived and symbolic dimensions. In the context of urban health, mental maps are useful, for example, to study what kind of spatial features and spatialized meanings encourage or discourage healthy activities: Wridt has used a combination of mental mapping and GIS to uncover what spatial features and meanings influence children’s physical activity in a low-income neighborhood [61]. They are also useful to discover the subjective boundaries of neighborhoods [60]. This is an important task: researchers often use official and/or arbitrary delimitations of neighborhoods, which may be detached from the definitions of place that are meaningful to locals [62]. Mental mapping may also highlight how different residents may define the same neighborhood differently, revealing the superimposition of multiple meaningful neighborhoods [60].

### Group Interaction Methods

Some methods, such as focus groups and the world café, generate data by promoting interaction between research participants. A focus groups is a guided discussion between a relatively small number of participants. It is not a collective interview: participants are encouraged to discuss with each other and spontaneously intervene in the conversation, instead of simply answering questions put by the moderator [63–65]. The interactional nature of the method generates a range of views [66, 67] and may uncover unforeseen nuanced complexities [66]. Individual accounts are not independent from each other neither from the group [68]. The group dynamics at work might contribute to generate consensus and new ideas [63, 66]. Participants may collectively work on complex ideas, while the interaction may generate tensions that influence responses and how ideas and consensus emerge [66]. Therefore, some authors argue that focus groups are not ideal to gather information on individual experiences and perspectives [63]. Nevertheless, in practice, focus groups are often employed to collect individual data. Researchers have used focus group data at three different levels—individual, group, and interactive. The first refers to the individual views and experiences revealed during the interaction, which are frequently complemented with data collected by other means [66]. The group level allows to explore agreement and disagreement [66], tension and ambivalence [67], and is often used to develop and test other data collection instruments [66]. Finally, the interaction level is used to analyze interaction [67], build hypothesis and conduct exploratory research [66].

The world café promotes collaborative dialogue among a larger number of participants. The discussion takes place in several rounds, during which participants are differently distributed in small groups. Each group may appoint a host that will stay at the table when a round ends, while the other group members change places to continue the conversation, thus linking many smaller conversations to a broader one [69, 70]. After several rounds of discussion within the rotating small groups, the whole group shares its insights and discoveries [70], enabling knowledge exchange, collective learning and cross-pollination of ideas [69, 70]. Therefore, it is well-suited to generate new ideas [71]. It may also promote the emergence of networks between participants [72].

Accounts generated by focus groups and similar methods are different from those generated from interviews, as they are of a more public nature [64]. Like interviews, group discussions are interactions and generate verbal data. Therefore, most of the debates about interviewing that we mentioned earlier are also relevant [34], perhaps even more so, given how the group composition and interaction shapes individual accounts.

These methods might be used in multiple ways in urban health research, namely to generate insights on how relevant groups (for example, city planners) shape the urban environment and collectively talk about health-relevant topics, to gather insights on relevant issues from different groups of stakeholders (including key informers and “regular” city inhabitants), or to generate new ideas about those issues. For example, Bhuyan and others [73] combined interviews with key informants with focus groups with older residents in an exploratory study about age-friendly neighborhoods. They identified what features a neighborhood should have in order to be age-friendly, from the perspectives of participants. On another example, Rivera-Navarro and co-authors [74] studied the influence of local food environments on dietary behavior according to the accounts of residents of three Madrid neighborhoods, using interviews to examine individual perceptions and focus groups to investigate collective perceptions among residents of each neighborhood.

### Qualitative Methods Combined With Geographic Information Systems (GIS)

Qualitative methods can be combined with GIS, as we have seen concerning mental mapping. This combination provides nuanced and contextual information about how people relate with place [48]. GIS may be used to add a cartographic dimension to go-along interviews. Doing so spatially contextualizes the data generated by the interviews, and links meaning to precise points in space [75]. For example, in a study on substance use among LGBTQ+ young adults [76], researchers logged locations and smoking behaviours during 30 days using GPS and a smartphone app to identify participants’ most frequent smoking locations/times, then interviewed participants to explore experiences and meanings of smoking locations and practices. The findings may be useful to inform tailored tobacco interventions. GIS may be also used with photovoice, adding a spatial dimension to the photographs obtained by participants and allowing to link space, images and meaning. This has been used to study gentrification [77], a phenomenon that, as mentioned above, is relevant to health.

### Analysis

There are many qualitative methods of data analysis. We cannot cover them exhaustively. Within several of them, different approaches exist. For example, consider two versatile and widely used methods, thematic analysis and qualitative content analysis. There are several different approaches to the former, some of which may be quite similar to some of the multiple versions of the latter. Thus, some authors argue that differences between both are mainly an issue of context or semantics [78].

There is no ideal method of qualitative data analysis: deciding how to analyze data is a process guided by the kind of data gathered, the goals and general outline of the research and by the researcher’s epistemological and theoretical leanings. While some methods seem adaptable to multiple theoretical and philosophical inclinations, either because multiple versions exist or they are presented as atheoretical, others are closely tied to specific philosophies and theories [78]. Thematic analysis and qualitative content analysis are generally examples of the former, as they have been used under various theoretical and epistemological orientations [78–80]. Both are concerned with description and, to a variable degree across different versions, interpretation of meaning. Conversely, the constant comparative method [14, 81] was developed within grounded theory to assist in the development of concepts and theories. Different methods have different goals and analytical focuses, while dealing mainly with textual data (sometimes also images and audiovisual material) and focused on achieving an interpretive understanding unrelated to measurement and quantification. While some analytical methods may be used with computer assisted qualitative data analysis software, others may not.

Documents often contain important information about urban context, urban policy and health policy, and may also contribute to shape these [82]. Thus, we briefly discuss qualitative document analysis, a term encapsulating a broad set of approaches to the selection, analysis and sometimes evaluation of pre-existing documents (including digital files and audio-visual material) for research purposes [83, 84]. Qualitative document analysis is a broad descriptor [85] that overlaps with other qualitative analytical tools, since many techniques (such as, for instance, qualitative content analysis) may be used as part of systematic and reflexive analyis of documents [82–85], in accordance with the goals and the theoretical and epistemological assumptions guiding the research [86]. With the growth of digitalization, internet use and computer research tools, more documents become available and retrievable, increasing the relevance of this method [87]. Document analysis may yield data, namely about context, that complements those collected with other methods [85, 86]. Documents may be analyzed as sources of research data or as objects of inquiry themselves [87]. In the first case, their contents may be seen as evidence; in the second case, document analysis may be used to study broader policy options or to unveil discourses or ideologies [86]. Either way, documents are not neutral containers of data: they are socially situated. Researchers should be aware of their context of production and function, as well as consider the relationships between production, consumption and content of documents [88]. Moreover, documents may tell more about their authors’ intentions than the issue they refer to [86], and their content is meant to suit the purposes of their authors, not of researchers [83]. Inspecting a document while considering its context may contribute to evaluate its authenticity, credibility, representativeness and meaning [84]. Dalglish and others [89] suggest a 4-step approach to document analysis in health policy research: reading the documents, extracting the data, analysing the data and distilling the results.

Considering the range of potentially relevant documents for the inquiry of urban health, from newspaper articles to planning documents and official reports from local and central governmental agencies and other organisations, this method may be valuable for urban health researchers. For example, Macassa and others [90] have interviewed key stakeholders and analyzed official local climate change adaptation plans to understand how different coastal cities accommodate public health concerns in local adaptation.

### Qualitative Sampling Strategies

Qualitative sampling is seldom oriented by probabilistic principles, rendering considerations about population representativeness and other statistical criteria often irrelevant. Samples are usually comparatively small and meant to be studied intensively [91]. Consequently, generalizability, when applicable, has a non-probabilistic meaning: making general statements from a limited number of known cases [92], often by linking results to theory [92–95]. In this sense, generalizing qualitative results is possible from small, non-probabilistic samples. One should be aware that generalization is not a consensual goal among qualitative researchers and that it might even be incompatible with some ontological and epistemological standpoints [96].

There are different sampling strategies, which may vary across qualitative research approaches [91, 96–98]. Even the definitions and terminology used might be variable or inconsistent across different research traditions [97]. However, it is desirable to select “information-rich cases,” i.e., cases that might offer rich opportunities for insight on the phenomenon being investigated. This is the central idea of purposive sampling, as influentially discussed by Patton [98].

Samples might not be always rigidly defined early on. Instead, they may evolve during the course of the research [97]. An example of this is theoretical sampling, a method developed under grounded theory where the sample is progressively defined while guided by the theory being built from the data [14]. However, due to practical reasons related to project approval, funding securing and budgeting, researchers using this strategy may need to estimate a sample size on the onset. The concept of saturation, also from grounded theory, often plays an important role in gradual sampling processes. In its simplest and most general sense, saturation refers to a point where further data collection and analysis no longer lead to new relevant insights [99]. Instead of using a sample with a pre-defined size, the researcher stops adding cases when additional data becomes redundant. While saturation is influential to the point of being sometimes labeled a “gold standard” for defining sample size in qualitative health research [100], it is also a contested concept [101, 102] that encloses different understandings and operationalization strategies [103, 104].

Table 1 summarizes the discussed data collection and analysis methods.

**TABLE 1:** Summary of discussed methods (Porto, Portugal, 2024).

Type of method	Variants	General purpose of the method
Observation	Non-participant	Study practices and behaviours in their context
	Participant	
	Shadowing	
Interviews	Semi-structured interviews	Generate detailed accounts of experiences, practices and meaning, possibly including a temporal and/or spatial dimension
	Go-along interviews	
Group interaction methods	Focus Groups	Study group dynamics
	World Café	Generate new ideas
		Promote collective discussion and reflection
Visual methods	Photovoice	Discuss meaning through pictures
Participatory research		Promote collective discussion and reflection
Action research		Empower participants and promote change
Mapping	Mental mapping	Study representations and meanings of space
Analysis	Document analysis	Reviewing, analyzing and evaluating documents as sources of data or as research objects themselves

## Discussion

There are published designs of qualitative urban health studies, such as [6], but this is, to the best of our knowledge, the first review discussing qualitative methods for urban health research.

Qualitative methods are flexible [8, 17, 23] in design and implementation: they may accommodate and explore unforeseen data. This contrasts with the high level of standardization of quantitative methods. They deal with rich, detailed and mostly non-numerical data, generally in the form of text, but possibly also images, objects or audio-visual records. Qualitative research has also been used to inspect causality, generally subscribing to a weaker understanding of causality [105].

Qualitative methods are generally well-suited to investigate perceptions in-depth, as well as the meaning attached to contexts, practices and experiences [8, 17, 106]. Their ability to gather nuanced data allows the investigation of complexity, ambiguity and contradiction [8]. They allow to study how structural and contextual forces act and to study practices and behaviors in context [6, 74]. They may contribute to improve our knowledge about how urban dwellers make sense of the urban environment and interact with it, how they change that environment while it also shapes their actions, a process mediated by their position in the social space and associated resources. This is crucial to understand the urban environment as a health determinant.

We have presented qualitative methods as tools that may be employed to meet specific research goals, while downplaying their philosophical underpinnings. This is a pragmatic (in the sense of oriented to “what works”) stance also advocated by others [18, 107]. It assumes that methods are not too strictly connected to epistemologies [107], and that methodological issues, more than epistemological issues, are paramount to method selection [18]. However, it is important to recognize that, beyond this stance, different philosophical leanings, each involving certain ontological and epistemological positions, coexist among qualitative researchers [105, 108]. These philosophies influence, implicitly or explicitly, how researchers think of methods, data and the research process. For example, some of the criticisms directed at the interview as a tool to inspect individual experiences and views that we mentioned earlier are informed by certain versions of constructionism [37, 38], a set of related philosophical standpoints generally arguing that any apparent reality is the object of selection and construction processes [105]. Constructionism also questions the notion of generalizability [96]. These are contentious issues that fall out of our scope, but important ones to acknowledge.

The pragmatic stance we adopt here has also been used to legitimize the combination of qualitative and quantitative approaches, drawing on the arguments that 1) the differences between them are not incommensurable and 2) since each approach can provide a vantage point that the other cannot, combining both provides more complete results [107, 109]. Mixed methods practitioners have been proposing specific frameworks to combine both types of methods [109, 110], and advancing pragmatism (in the sense of a philosophical tradition) as the philosophical underpinning of mixed methods research [111, 112].

We should discuss the limits of qualitative methods. They are ill-suited for measurement, for establishing general trends and associations and for probabilistic generalization. Moreover, qualitative research is home to multiple research traditions and philosophies that may sometimes present concepts and ideas conflicting in some aspects while overlapping in others. Consequently, there are conflicting views about the uses of specific data collection and analysis techniques. This plurality might be challenging, especially for researchers trying to find their feet on the field. It may also complicate the appraisal of qualitative studies [13]. Moreover, although their flexibility is a strength, the general lack of standardization and multiple possibilities concerning choice, design and implementation of methods requires solid theoretical and methodological training [23] and may also be challenging for novice qualitative researchers.

### Conclusion

Qualitative methods provide valuable and flexible ways to improve our collective understanding of urban health. They allow to observe, in-depth, phenomena from a different vantage point than quantitative methods, one closer to everyday experience and its contextual meaning. This is important to improve our general knowledge about cities and health, and is also to inform decisions concerning urban and health planning. Furthermore, some qualitative methods provide opportunities to involve the citizens in such decisions. Therefore, qualitative methods are a valuable set of tools for urban health researchers.
